# 11.M. Workshop: Vaccination hesitancy in the Western Balkans and inclusive governance

**DOI:** 10.1093/eurpub/ckac129.734

**Published:** 2022-10-25

**Authors:** Organised by: EURO HEALTH GROUP (Denmark), School of Public Health & Health Management (Serbia), Chair persons: Michele Gross (Denmark), Ulrich Laaser (Germany)

## Abstract

Vaccine hesitancy has been identified as one of the crucial contributors to the global decline in vaccination coverage for several well-established vaccines in previous decades and is listed by the World Health Organization as one of the top ten threats to global health. In developing countries, the leading reasons for under-vaccination appear to be the lack of access, low education and socio-economic status. In developed countries psychological, social, and contextual factors are defined as main drivers of vaccine hesitancy. With the COVID-19 pandemic the threat of vaccine hesitancy has become more evident and is now in the focus of strategies and efforts to improve and strengthen the interventions to combat vaccine hesitancy and increase vaccination coverage. Despite the availability of multiple effective vaccines against COVID-19, only 40% of population of Wester Balkans in average has been completely vaccinated (with a complete initial protocol), which is far below the world average (56%). Vaccine hesitancy largely jeopardizes the achievement of heard immunity, postponing the end of the pandemic. To explore reasons of vaccine hesitancy, the cross-sectional and quasi-experimental studies were performed from July to September 2021 in five countries of the Western Balkans: Albania, Bosnia-Herzegovina, Montenegro, North Macedonia, and Serbia. The results will be presented and discussed from a policy perspective. The first presentation will introduce to the actual debate around vaccination hesitancy; the second presentation describes the survey design and key results related to societal factors (confidence in political and health authorities, science, and pharmaceutical companies), social responsibility (personal sense of responsibility in achieving collective immunity and contagion prevention) and the credibility of information sources about COVID-19 vaccines; the third presentation will discuss components of credibility (expertise, trustworthiness, and caring/goodwill) after respondents’ exposure to messages with narratives on COVID-19 vaccine decisions. The fourth presentation is going to identify promising policy options. The objective of the workshop is to present and discuss the results of the Western Balkans study, relate them to corresponding studies in Europe and beyond, and propose effective interventions. The audience is invited to discuss the findings and the practicability of strategies to improve the acceptance of qualified information in the population, how to enhance trustworthy, how to improve vaccination rates. The discussion will focus on the following questions: a) What are the main drivers of vaccination hesitancy? b) Which messaging format is most convincing and trustworthy? c) Why is the global dimension essential?

**Key messages:**

• Vaccine hesitancy is a serious threat and reason for the insufficient vaccination coverage and suboptimal herd immunity in the Western Balkans.

• Vaccination policy has to enhance information trustworthy and source credibility at the national level and argue for fair resource distribution in the global dimension.

